# Sustainable Assessment of Bio-Colorant from Bakain Bark (*Melia azedarach* L.) for Dyeing of Cellulosic and Proteinous Fabric

**DOI:** 10.3390/molecules29184392

**Published:** 2024-09-15

**Authors:** Fatima Batool, Maheen Fatima, Shahid Adeel, Sheikh Asrar Ahmad, Md. Reazuddin Repon, Aminoddin Haji

**Affiliations:** 1 Department of Botany, Division of Science and Technology, University of Education, Lahore 54770, Pakistan; fatima.batool@ue.edu.pk; 2 Department of Chemistry, University of Education Lahore, Vehari Campus, Vehari 61100, Pakistan; maheen9293@outlook.com (M.F.); asrarchemist@ue.edu.pk (S.A.A.); 3 Department of Applied Chemistry, Government College University Faisalabad, Faisalabad 38000, Pakistan; 4 Department of Textile Engineering, Daffodil International University, Dhaka 1207, Bangladesh; reazmbstu.te@gmail.com; 5 Department of Production Engineering, Faculty of Mechanical Engineering and Design, Kaunas University of Technology, Studentų 56, LT-51424 Kaunas, Lithuania; 6 Department of Textile Engineering, Yazd University, Yazd 8915818411, Iran

**Keywords:** tannin, bio-mordant, bakain bark, cotton, silk

## Abstract

The current study proceeded to reduce the environmental hazards spreading worldwide due to synthetic dyes. To overcome these problems, eco-friendly natural dyes are introduced as alternative sources of synthetic dyes. The present study was focused on exploring the bio-colorant of the aqueous and acidic extract of the bark of *Melia azedarach* L. for the dyeing of both silk and cotton samples. The results of the extraction medium specified that the aqueous extract gave maximum colorant solubility and upon fabric dyeing produced higher color strength in contrast to the acidic medium. The optimization experimentation data showed that excellent color strength of silk fabric was found at 45 min dyeing time duration, in 35:1 mL dye extract, and using 2% salt (NaCl) as an exhausting agent, whereas cotton fabric showed the maximum K/S value at 60 min dyeing time, in a 45:1 mL liquor ratio, and with the use of 2% salt. Bio-mordants produce different shades on both fabrics. Bio-mordanting experiments on silk revealed that pre-mordanting with 2% turmeric and 3% pomegranate, and post-mordanting using 3% turmeric and 2% pomegranate produced a darker shade. In the case of cotton, the pre-mordanted samples with 2% turmeric and 3% pomegranate and the post-mordanted samples with 4% turmeric and 4% pomegranate gave the highest color strengths. All the mordanted samples gave excellent fastness ratings. Overall, it has been found that Bakain bark proved to be an excellent source of tannin. The result of this study showed that it could be a cost-effective and eco-friendly dye source for textile progress.

## 1. Introduction

Plants have been used for various purposes, such as lumber, fuel, food, and medicine, for the benefit of mankind since prehistoric times [1,2]. Another vital function is that the secondary metabolites of plants, such as phenolics, betanin, tannin, carotenoids, rutin, and anthocyanin which are present in different parts of the plant, have also been used as colorants since ancient times [3]. After the discovery of the chemical sources of dyes, plant-based colorants considerably declined for coloration purposes [4]. Synthetic dyes are mostly produced from petrochemical sources; however, synthetic colorants are toxic, non-renewable, and non-biodegradable [5]. Therefore, it generates several types of pollutants during manufacturing and textile processing, which harshly damage the environment, aquatic ecosystems, biodiversity, and human health [6]. Water pollution is the most common due to synthetic colorants because the effluents of synthetic colorants contain vast amounts of dyes, hazardous and toxic chemicals, auxiliaries, and additives [5]. Most effluents of synthetic colorants are composed of various chemicals such as acetic acid, sulfur, nitrates, naphthol, chromium, vat dyes, soap, and heavy metals. They enter water bodies through industrial effluent that changes the quality of water, which is very dangerous for water life, human life, and agricultural land [7,8]. In aquatic ecosystems, textile effluent also decreases photosynthetic activity by disturbing light penetration, which directly affects the food chain [9]. In this way, synthetic colorants and their waste are dangerous for life. Moreover, due to strict environmental legislation, several agencies and countries, such as Germany, India, the USA, and the European Union, ban the utilization of synthetic colorants in food, medicine, and textiles for coloration purposes [10,11,12]. Therefore, there is a great demand for the exploration of alternate sources of synthetic colorants or the re-emergence of natural dyes for coloration purposes.

Natural colorants are biodegradable, non-allergic, and nontoxic [13,14]. Natural colorants were considered alternate sources of synthetic colorants and obtained from a variety of natural sources such as plants, animals, minerals, microbes, algae, and insects [3,15]. Furthermore, natural colorants also have valuable properties such as antioxidant, antibacterial, deodorizing, insect repellency, and UV protective properties [16,17]. Therefore, recently, the utilization of natural colorants has increased as a pH indicator in food coloration, textile coloration, histological staining, cosmetics, and the pharmaceutical sector [13,18]. Mordants are widely used in dyeing, and their essential task is to fix the colorant on the fabric surface by generating a chemical reaction between the fabric and dye molecules [19]. As a result, it improves the fastness properties with shade variation and also enhances the dye exhaustion rate [20,21]. Mordants commonly used in the mordanting process are metallic and synthetic sources [12,22]. However, the extensive application of these mordants in textile industries produced huge amounts of effluent, creating serious environmental problems. On the other hand, bio-mordants obtained from natural sources are sustainable, eco-friendly, easily available, and cost-effective [23,24,25,26]. Therefore, bio-mordants were used in this study.

*Melia azedarach* L., commonly known as Dhareek in Pakistan, belongs to the Meliaceae family [16]. According to phytochemical analysis, Bakain bark comprises different phytochemicals, including kulinone, melanin, tetrahydroxy-2-methyl anthraquinone, β-pinene, 12-acetoxyamoorastatin, galactopyranoside, fraxinellone, amoorastatin, kulactone, 12-hydroxyamoorastatone, methylkulonate, alpha-terpineol, kulolactone, and alpha-terpinene [27,28,29]. Moreover, it also contains flavonoids, polysaccharides, tannin, triterpenoids, steroids, and phenols having antioxidant, antiviral, antibacterial, anti-feedant, and insecticidal activities, and in China, it is mostly used in Chinese traditional medicine [30,31,32]. Previously, different plant barks have been used as natural dyes [4]. No literature studies have been found on the exploration of Bakain bark dye in an aqueous medium and its application on both cotton and silk fabrics.

The current research work is focused on the utilization of Bakain bark as a natural dye source. The objectives of the study are to explore the colorant potential of Bakain bark, which is employed for fabric dyeing, during the transformation of waste material into valuable products and the use of pollution-free bio-mordants to develop different shades.

## 2. Materials and Methods

### 2.1. Materials

**Figure 1:**
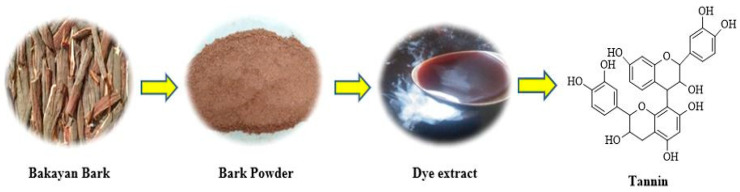
Preparation of Bakain bark dye extract.

Bakain (*Melia azedarach* L.) bark was collected from Hasilpur, Pakistan. The plant material (Bakain bark) was washed, dried, and crushed into a powder before being used to extract natural colorant (Figure 1) for dyeing silk (GSM = 40 g/m^2^) and cotton fabrics (GSM = 70 g/m^2^). Both fabrics were purchased from the textile market of Faisalabad, Pakistan.

### 2.2. Extraction of Bio-Colorant

Bio-colorants from plant bark were extracted by the use of two extraction media: aqueous (tap water) and acidic (vinegar 4%). The extraction was accomplished by boiling 5.0 g of Bakain bark powder in 100 mL of tap water for 40 min on a hot plate. A similar procedure was employed in the case of 4% acidic extraction. After boiling, filtrate was applied to cotton and silk fabrics at the Chemistry Lab at the Department of Chemistry, University of Education, Vehari Campus.

### 2.3. Optimization of Different Parameters

Various dyeing parameters were optimized to attain one of the best dyeing conditions. The time durations of 15, 30, 45, 60, and 75 min; material–liquor ratios of 25:1, 35:1, 45:1, 55:1, and 65:1; and salt concentrations of 1, 2, 3, 4, and 5% were used [33].

### 2.4. Bio-Mordanting

Herbal bio-mordants pomegranate peel (*Punica granatum*) and turmeric (*Curcuma longa*) in 1–5% were used as pre- and post-mordants under optimized dyeing conditions to generate a diverse spectrum of color shades. The extraction of bio-mordant was performed by the process described by Mahboob [34].

### 2.5. Computation of K/S Value and Fastness Attributes

Dyed fabrics were observed by Spectra Flash (SF-600) for the observation of the color strength (K/S) value at the Department of Chemistry, Government College University, Faisalabad. K/S was calculated based on the Kubelka–Munk theory mentioned in Equation (1). The bio-colorant stability of the tested samples against washing, light, and rubbing was reported by standard protocols such as ISO 105 B02 [35] protocol for light fastness, ISO 105 X-12 [36] for rubbing fastness, and ISO 105-C03 [37] for washing fastness.

(1) $$\frac{K}{S}=\frac{{\left(1-R\right)}^{2}}{2R}$$

where *R* is the reflectance of incident light from the dyed materials, and K and S are the absorption and scattering coefficients of the dyed fabric, respectively.

## 3. Results and Discussion

The current study was performed to isolate the bio-colorant of the Bakain bark by employing aqueous and acidic media and used for silk and cotton dyeing. The extraction experimentation details (Table 1) suggest that an aqueous medium gives maximum colorant yield, possibly due to the colorant molecules more effectively dissolving in an aqueous medium than an acidic medium. The current results are consistent with the previous findings that confirmed a higher extraction of plant secondary metabolites in an aqueous medium in comparison to an acidic medium [38]. Hence, the aqueous medium is found to be more appropriate and cost-effective for the extraction of dyes from the Bakain bark, while the acidic medium failed to give the maximum colorant yield. Both the aqueous and acidic Bakain bark dye extract gave varying trends in color strength onto dyed fabrics. The silk and cotton dyeing results revealed that the aqueous Bakain bark dye extract produced a higher K/S value on both fabrics.

**Figure 2:**
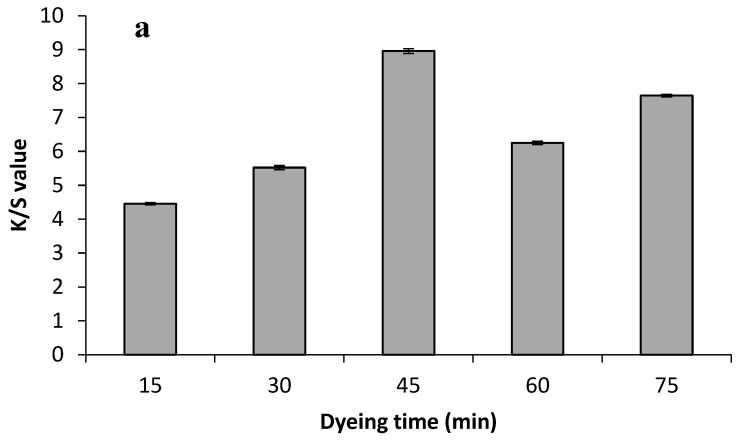

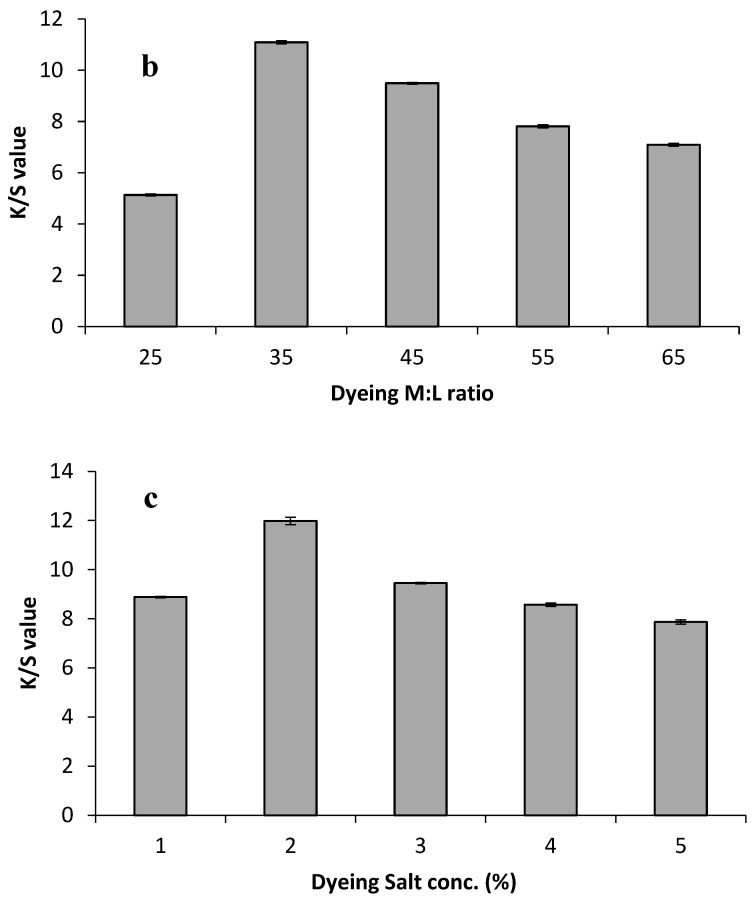
Impact of varying dyeing levels: (**a**) varying dyeing time; (**b**) varying material to liquor ratio; (**c**) varying salt level on the K/S value of the dyed silk samples in the aqueous dye extract of Bakain bark.

**Figure 3:**
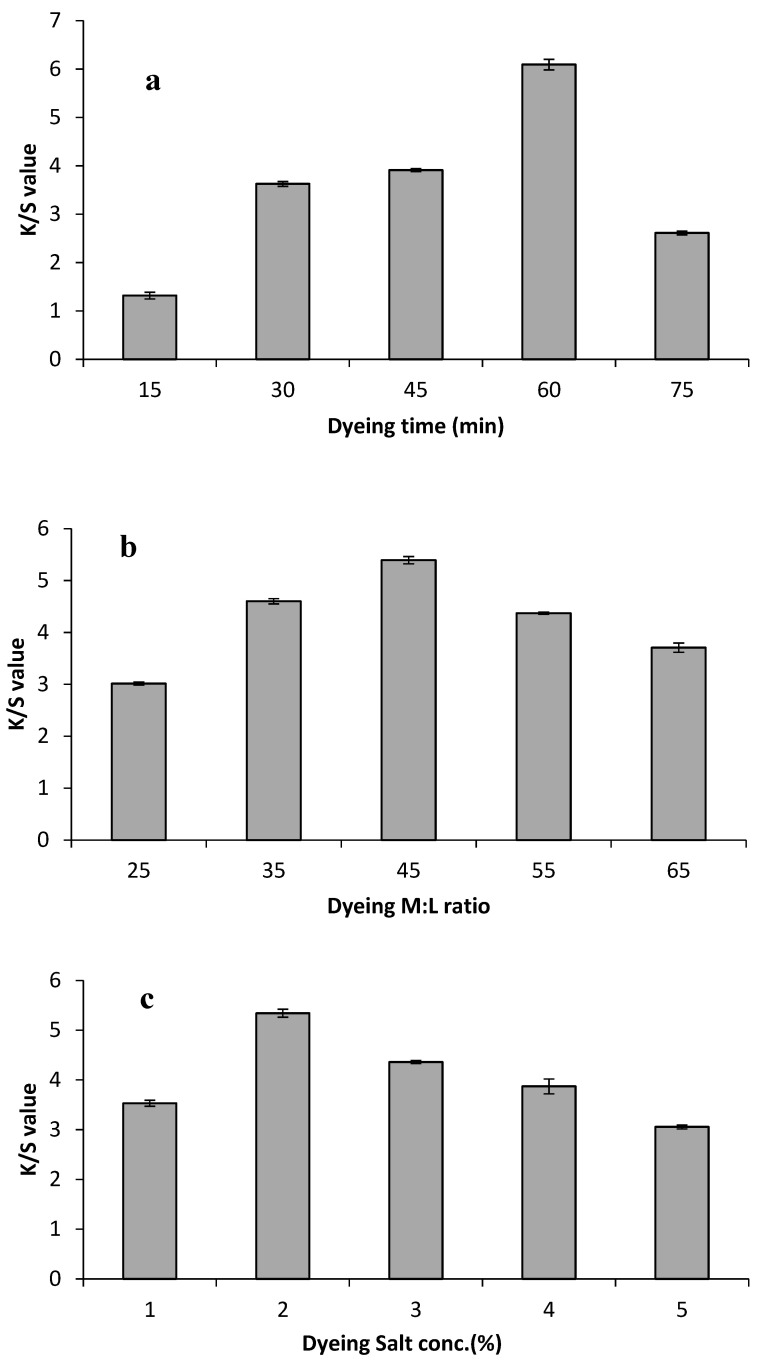
Impact of varying dyeing levels: (**a**) varying dyeing time; (**b**) varying material to liquor ratio; (**c**) varying salt level on the K/S value of the dyed cotton samples in the aqueous dye extract of Bakain bark.

Optimal dyeing conditions, including dye extract ratio, dyeing time, and salt concentration have a functional role in natural dyeing. The given result (Figure 2a and Figure 3a) revealed the role of various time durations on the color strength value of both Bakain bark-dyed fabrics. Color strength was maximized by a rise in time duration; therefore, 45 min dyeing duration for silk fabric and 60 min dyeing time duration for cotton proved to be the optimized dyeing level to obtain higher color strength value on the fabrics than the other dyeing durations. An increase in dyeing time leads to a rise in the kinetic energy of the dye molecules and causes maximum dye sorption on the silk and cotton samples. However, the color strength value decreased beyond the optimal dyeing conditions due to a disruption in the dyeing bath equilibrium or desorption of colorant molecules on the dyed sample [20]. The material–liquor ratio of the dye extract plays an important role in dyeing. The given results (Figure 2b and Figure 3b) showed the role of variable dye extract levels on both the dyed fabrics. The results demonstrated that a small dye extract volume gives low colorant yields due to fewer dye molecules in the lower dye volume ratio while too much dye extract volume also disturbs colorant yield due to the dye molecules starting to aggregate with each other, resulting in low color strength on fabric [20]. Hence, 35 mL for the silk fabric and 45 mL for the cotton fabric gave the maximum K/S value on the Bakain bark-dyed fabrics.

Salt in the dyeing process continually assists in achieving the highest exhaustion of colorant because it plays a role in moving the colorant molecules from the extract solution toward the fabric surface within a short range of attractive forces [39]. In the current studies, the results shown in Figure 2c and Figure 3c indicated the impact of various salt levels on the dyed silk and cotton fabric. It showed that the application of 2% salt during the dyeing process significantly increased the exhaustion of colorant molecules on both silk and cotton, and the results gave maximum color strength. Overall, 2% salt proved to be cost-effective in attaining excellent results, whereas excess salt level leads to the desorption of the bio-colorant and yields minimum K/S value to the dyed fabrics.

**Figure 4:**
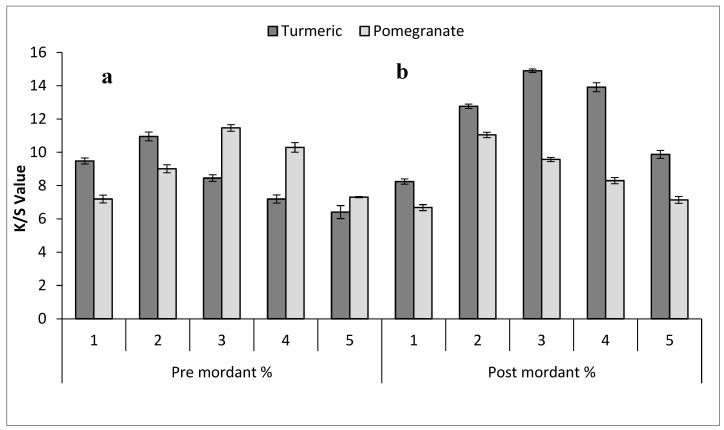
Effect of bio-mordants on the K/S value of (**a**) pre-mordanted and (**b**) post-mordanted silk dyed in the aqueous dye extract of Bakain bark.

**Figure 5:**
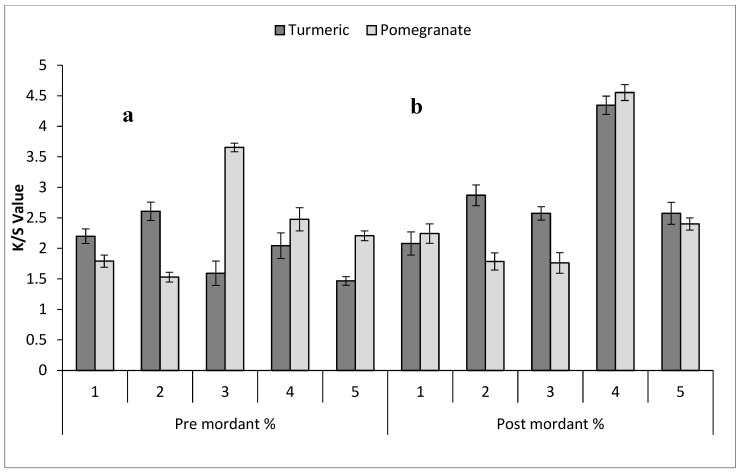
Effect of bio-mordants on the K/S value of (**a**) pre-mordanted and (**b**) post-mordanted cotton dyed in the aqueous extract of Bakain bark.

Bio-mordanting is a crucial step in natural dyeing to attain different shades on the dyed fabrics [40]. Previously, various herbal bio-mordants have been employed in natural dyeing [23,24,40]. In the current study, turmeric and pomegranate in varying levels are being used to improve fastness properties and to develop a broad range of shades (Table 2) with higher color strength (Figure 4 and Figure 5). Pre-mordanting enhances color vibrancy and consistency by preparing the fiber to bond well with the dye. Post-mordanting can deepen or modify colors by interacting with the dye already bonded to the fiber, useful for adjusting shades or improving color fastness post-dyeing. Turmeric (curcumin) and pomegranate (tannin) are selected as mordants for their abilities: Turmeric can impact dye color, often adding yellow tones or intensifying certain hues. Pomegranate’s tannins can deepen dye colors, expanding the range of shades achievable in the fabric. The result (Figure 4a) showed that pre-mordanted silk with 2% turmeric (curcumin) and with 3% of pomegranate (tannin), and in silk bio post-mordanting, 3% turmeric (curcumin) and 2% of pomegranate (tannin) gave a darker shade with maximum color strength (Figure 4b). In cotton, bio-mordants also produced an array of color shades. The cotton bio-mordanting results (Figure 5a,b) indicated that bio pre-mordanting with 2% turmeric (curcumin) and with 3% pomegranate (tannin) gave a higher K/S value with a darker shade. Meanwhile, 4% turmeric and 4% pomegranate application on the dyed cotton sample using an aqueous extract of Bakain bark gave excellent results with maximum color strength value. The result showed that the acceptable color strength of using bio-mordants on fabrics might be due to the presence of tannin in pomegranate and curcumin in turmeric, which formed a strong H bonding with the tannin of the colorant molecule and with the –OH group of cotton as well as with the amide unit of the silk sample and produced a dark shade with excellent color strength [18]. Hence, 3% pomegranate as pre-mordant and 3% turmeric as post-mordant are optimal levels for silk dyeing with Bakain bark dyes. In the case of cotton, 3% pomegranate peel as a pre-mordant and 4% pomegranate peel as a post-mordant produced excellent results on the fabric during the dyeing process. Comparatively, silk produced darker shades with higher K/S values.

Bio-mordanting also improved the fastness properties of the dyed fabrics. Fastness results (Table 3) showed that the optimal bio-mordanted silk and cotton samples produced excellent fastness ratings. The fastness properties of the mordant-treated fabrics might be due to the additional H-bonding presence between the surface of the fabric, and the functional groups of mordant and dye molecules [18,40]. Variations in the fastness characteristics of the mordanted samples dyed in natural dyes might depend upon the mordant nature and the fabric type. Our results are consistent with the previous findings [41,42], which mainly describe that bio-mordants, including turmeric and pomegranate, have significantly improved the fastness characteristics of the cotton samples dyed in the natural colorant of plant residues. Hence, bio-mordant proved to be an excellent source for obtaining a wide range of color shades.

## 4. Conclusions

Bakain bark proved to be an eco-friendly source of colorant. The present study successfully discovered the extraction of bio-colorants from Bakain bark in aqueous (tap water) and acidic media and their application on silk and cotton fabrics. The aqueous extraction medium gave maximum colorant solubility and upon silk and cotton dyeing produced the best results with higher color strength. The optimization of the dyeing parameters for silk revealed that the fabric dyed for 45 min of dyeing time, with a 35 mL dye extract, and 2% of NaCl as an exhausting agent in an aqueous medium gave the highest color strength. However, in the case of cotton fabric, optimized dyeing parameters were 60 min of dyeing time duration, 45 mL dye extract, and 2% NaCl. The bio-mordanting experiments revealed that applying both bio-mordants, including turmeric and pomegranate, significantly improved the color strength value and shades of both fabrics. Furthermore, mordanted cotton and silk samples also revealed excellent colorfastness values. Overall, the results concluded that Bakain bark could be a promising source of tannin dyes for fabric dyeing.

## Figures and Tables

**Table 1:** Color strength value of dyed fabrics using Bakain bark dye extracted in both aqueous and acidic (vinegar) media.

Extraction Medium	Conc.%	(Silk Fabric) K/S Value	(Cotton Fabric) K/S Value
Tap water	100%	2.8213	2.0112
Acid (Vinegar)	4%	1.1932	0.2731

**Table 2:** Color shade of non-mordanted and bio-mordant treated fabrics dyed in Bakain bark dye extract.

Fabric Types	Mordanted Fabrics	Shade
Silk	Control sample	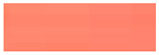
	Pomegranate peel 3% pre-mordanted silk	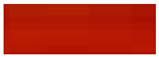
	Pomegranate peel 2% post-mordanted silk	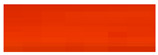
	Turmeric 2% pre-mordanted fabric	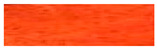
	Turmeric 3%post-mordanted fabric	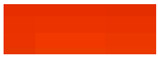
Cotton	Control	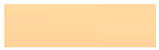
	Pomegranate peel 3% pre-mordanted cotton	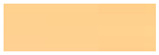
	Pomegranate peel 4% post-mordanted sample	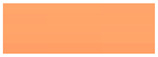
	2% turmeric pre-mordanted cotton	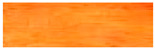
	4% Turmeric post-mordanted cotton	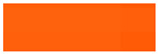

**Table 3:** Effect of bio-mordants on the fastness attributes of the Bakain bark-dyed fabrics.

Mordant	Mordant Ratio %	Turmeric			Pomegranate Peel		
		LF	WF	RF	LF	WF	RF
Pre-mordant (Silk)	1	4/5	4/5	3/4	3/4	3/4	5
	2	5	4/5	4/5	4/5	4/5	4/5
	3	4/5	4	3/4	3/4	4/5	4
	4	4	3/4	5	4/5	5	4/5
	5	3/4	4/5	4/5	3/4	3/4	4/5
Post-mordant (Silk)	1	3/4	5	4/5	4	3/4	5
	2	4/5	3/4	5	3/4	5	3/4
	3	4/5	4/5	4/5	4/5	4/5	3
	4	4	3/4	4/5	3/4	3/4	4/5
	5	3/4	4	3/4	4	4/5	¾
Pre-mordant (Cotton)	1	4/5	3/4	4/5	3/4	4	4/5
	2	4	5	3/4	4	3/4	5
	3	3/4	4/5	5	3/4	4/5	4/5
	4	4/5	3	4/5	4/5	4	4/5
	5	3/4	3/4	5	4/5	4/5	4
Post-mordant (Cotton)	1	5	4/5	3/4	3/4	4	4/5
	2	4/5	3/4	5	3/4	4/5	5
	3	3/4	5	3/4	5	3/4	4
	4	4	4/5	4/5	4/5	4	¾
	5	3/4	3/4	4/5	4	5	3

LF = light fastness; WF = washing fastness; RF = rubbing fastness.

## Data Availability

Data are contained within the article.
